# miR-17 inhibition enhances the formation of kidney cancer spheres with stem cell/tumor initiating cell properties

**DOI:** 10.18632/oncotarget.1901

**Published:** 2014-04-16

**Authors:** Zsuzsanna Lichner, Carol Saleh, Venkateswaran Subramaniam, Annetta Seivwright, Gerald Joseph Prud'homme, George Makram Yousef

**Affiliations:** ^1^ Department of Laboratory Medicine, and the Keenan Research Centre for Biomedical Scienceatthe Li Ka Shing Knowledge Institute; ^2^ Department of Pathology and Laboratory Medicine, University of Toronto, M5G 1L5, Canada

**Keywords:** Clear cell renal cell carcinoma, microRNA, Cancer stem cell- Tumor initiating cell, Epithelial-to-mesenchymal transition

## Abstract

Renal cell carcinoma (RCC) is an aggressive disease, with 35% chance of metastasis. The ‘*cancer stem cell’* hypothesis suggests that a subset of cancer cells possess stem cell properties and is crucial in tumor initiation, metastasis and treatment resistance. We isolated RCC spheres and showed that they exhibit cancer stem cell/tumor initiating cell-like properties including the formation of self-renewing spheres, high tumorigenicity and the ability to differentiate to cell types of the original tumor. Spheres showed increased expression of stem cell-related transcription factors and mesenchymal markers.  miRNAs were differentially expressed between RCC spheres and their parental cells. Inhibition of miR-17 accelerated the formation of RCC spheres which shared molecular characteristics with the spontaneous RCC spheres. Target prediction pointed out TGFβ pathway activation as a possible mechanism to drive RCC sphere formation. We demonstrate that miR-17 overexpression interferes with the TGFβ-EMT axis and hinders RCC sphere formation; and validated *TGFBR2* as a direct and biologically relevant target during this process. Thus, a single miRNA may have an impact on the formation of highly tumorigenic cancer spheres of kidney cancer.

## INTRODUCTION

The ‘*cancer stem cell’* (CSC) model suggests that a small subset of cancer cells possess stem cell properties and play a crucial role in tumor initiation, metastasis and resistance to anticancer therapy [1]. CSC populations have been identified and characterized in a number of solid tumors, including melanoma, breast, brain, lung, colon, prostate, pancreatic and colorectal cancers [2]. The characteristic features of CSCs include: 1) self-renewing (tested by sphere formation in serum-free defined media, SFDM); 2) high tumorigenicity in xenograft-based model and 3) ability to differentiate to the cell types of the tumor of origin [1, 3]. The ‘*clonal evolution’* hypothesis, on the other hand, states that every cell has equal chance to gain a mutation that provides it with selective advantage to out-compete the neighboring cell populations and initiate a tumor. Recently, the two hypotheses began to merge since the ‘*dynamic CSC model’* suggested that cancer stem cells can evolve from differentiated cancer cells by de-differentiation [4]. Epithelial-mesenchymal transition (EMT), triggered by the activation of TGFβ pathway, is an important way to induce CSC formation in the epithelial cells of breast- [5], colorectal- [6], gastric- [7] and prostate [8] cancers. Moreover, EMT was associated with increased expression of stem cell-related transcription factors and with increased tumorigenic ability [5, 9]. The TGFβ-EMT axis functions in pathological conditions such as fibrosis and cancer [10], and under physiological conditions, such as normal kidney development and homeostasis [11]. The CSC and EMT concepts were integrated in a model which proposes that the stationary CSCs activate the EMT program to be able to migrate, and eventually go through MET at the metastatic site [12].

RCC is an aggressive disease, with a 5-year survival rate of 71.8% (compared to 96% for prostate cancer). RCC has ~37% chance of progressing to metastasis [13], where the survival drops to ~9% [14]. Metastatic RCC is therapy-resistant. The average response rates to chemotherapy, radiotherapy and immunotherapy do not exceed 10%, and patients eventually develop therapy resistance [15]. Literature describing stem cells of kidney cancers is limited. Bussolati et al reported the isolation of CD105+ cancer stem cells from renal carcinoma [16] and tumor spheres enriched in CSC were isolated from a kidney cancer cell line [17]. However, the molecular mechanism underlying the formation and maintenance of self-renewing stem cells in kidney cancer remain to be elucidated.

miRNAs are required for the maintenance of normal pluripotent embryonic stem cells [18] and they were shown to regulate the generation of induced pluripotent stem cells from terminally differentiated somatic cells [19]. The role of miRNAs in CSC formation has been recently documented in a number of cancers [20], with several miRNAs implicated in the regulation of the TGFβ-EMT axis [21]. For example the ZEB1/2-miR-200 regulatory loop is a driving force of cancer metastasis and EMT and also operates in renal tubular epithelial cells [22].

In this work, we examine the hypothesis that miRNAs contribute to the acquisition and maintenance of stem cell/tumor initiating cell characteristics in kidney cancer. We isolated self-renewing spheres from RCC cell lines (RCC spheres), and proved that they are highly clonogenic in vitro and are highly tumorigenic in xenograft-based model. These spheres showed increased expression of stem cell-related transcription factors and mesenchymal markers suggesting that EMT is a contributor in gaining CSC features. We identified miRNAs that were differentially expressed between RCC spheres and their parental cell lines. Predicted targets of these miRNAs were enriched in the members of TGFβ pathway. We also show that miR-17 inhibition results in formation of cancer spheres. Finally, we demonstrate that TGFβ1 induces sphere formation and that TGFβ receptor 2 (*TGFBR2*) is a possible biologically relevant target of miR-17 in this process.

## RESULTS

### Isolation of cancer spheres with stem cell properties from kidney cancer cell lines

**Figure 1:**
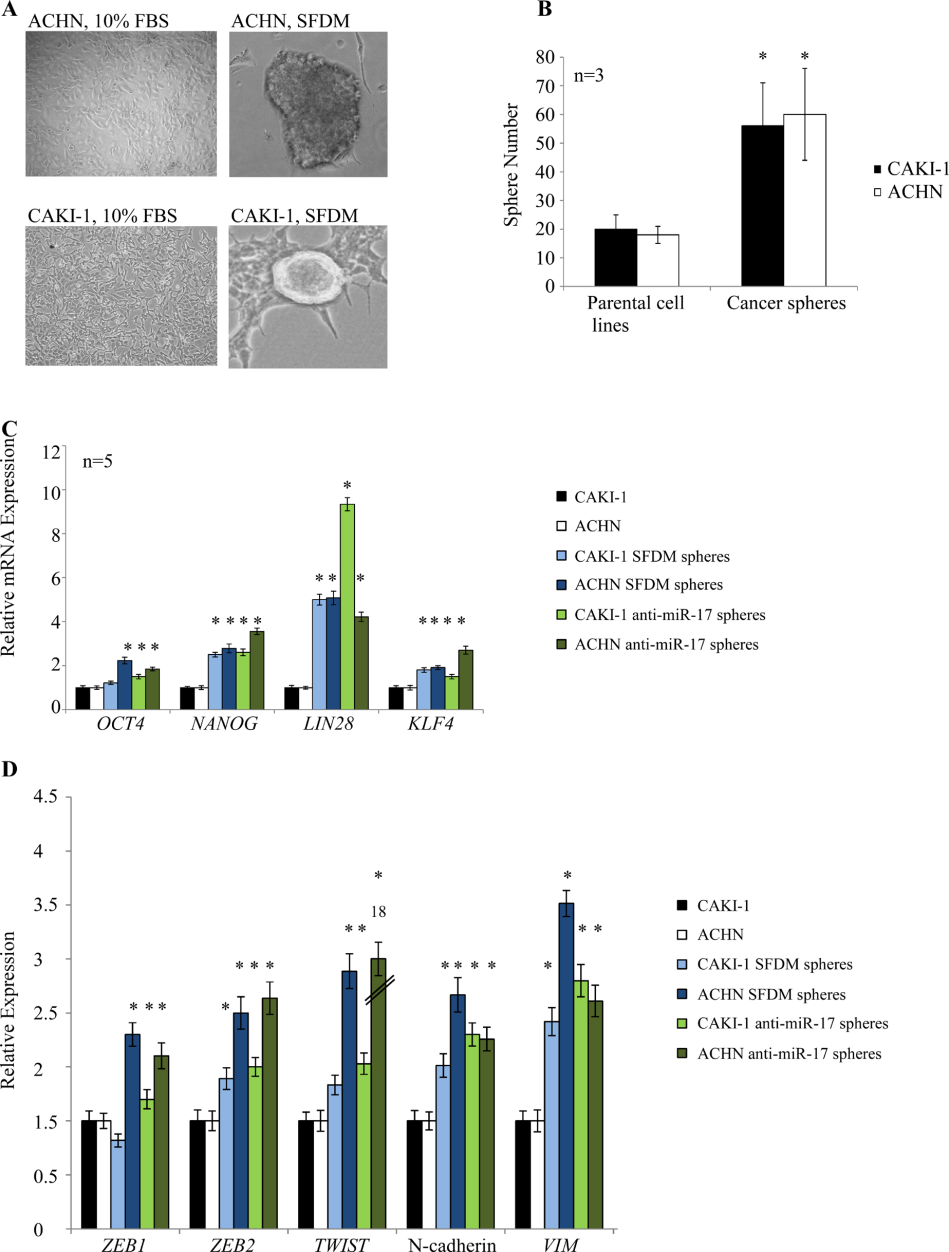
Kidney cancer cell lines form cancer spheres with stem cell-related features in serum-free defined medium (SFDM) and after anti-miR-17 transfection (A) ACHN and CAKI-1 cells were propagated in SFDM or transfected with anti-miR-17, leading to the formation of 3D RCC spheres that could be propagated by enzymatic dissociation. Cells kept in regular (10% FBS) medium did not form spheres. (B) Sphere formation assay compared the self-renewal capacity of the parental cell lines and their 3D sphere derivatives. RCC spheres showed significantly higher sphere forming ability. (C) Stem cell marker expression of the parental cells, RCC spheres established in SFDM and spheres established by miR-17 inhibition was quantified by RT-qPCR. (D) Mesenchymal marker expression of parental cells, RCC spheres established in SFDM and spheres established by miR-17 inhibition was quantified by RT-qPCR. VIM: vimentin, SFDM: serum-free defined medium

Kidney cancer cell lines derived from metastatic renal cell carcinoma (ACHN, CAKI-1) were cultured in SFDM which was shown to support the formation of CSC spheres. Under these conditions, we observed sphere formation from both cell lines (Figure 1A). Spheres could be propagated in three dimensional (3D) cultures in ultra-low adherent dish for several passages. When kept on adherent culture dish, spheres attached, spread and gave rise to a number of additional small colonies (Supplementary Figure 1A-C). Transferring to regular media (10% FBS) resulted in rapid differentiation of the spheres. Conditioned media did not initiate sphere formation.

The ability of cell lines to form non-adherent spheres in vitro depends on the presence of a self-renewing cell population [3, 23]. To assess the clonogenic potential of these sphere-forming cells, single cell suspension prepared from ACHN and CAKI-1 spheres and the parental cell lines was plated in SFDM. We observed large spheres in 4-6 weeks. The sphere-derived cells typically formed >2 fold more spheres than the parental cell lines (Figure 1B).

Next, we investigated if these spheres possess other stem cell-related characteristics. We first compared the expression of the *OCT4, NANOG, LIN28*, and *KLF4* stem cell transcription factors between the parental kidney cancer cell lines and their sphere derivatives. Our results indicated a significant 2-5 fold increase of all four stem cell markers in both ACHN and CAKI-1 spheres (Figure 1C). Stem cell induction, metastasis and dedifferentiation have recently been linked to epithelial-to-mesenchymal transition (EMT) in different cancers [5, 24]. To test whether a transition to mesenchymal traits was coupled with increased stem cell marker levels, we quantified the expression of a number of mesenchymal markers. We observed a significant increase in *ZEB1, ZEB2*, *TWIST1,* N-cadherin and vimentin expression in both ACHN and CAKI-1 derived spheres compared to their parental cells (Figure 1D).

**Figure 2:**
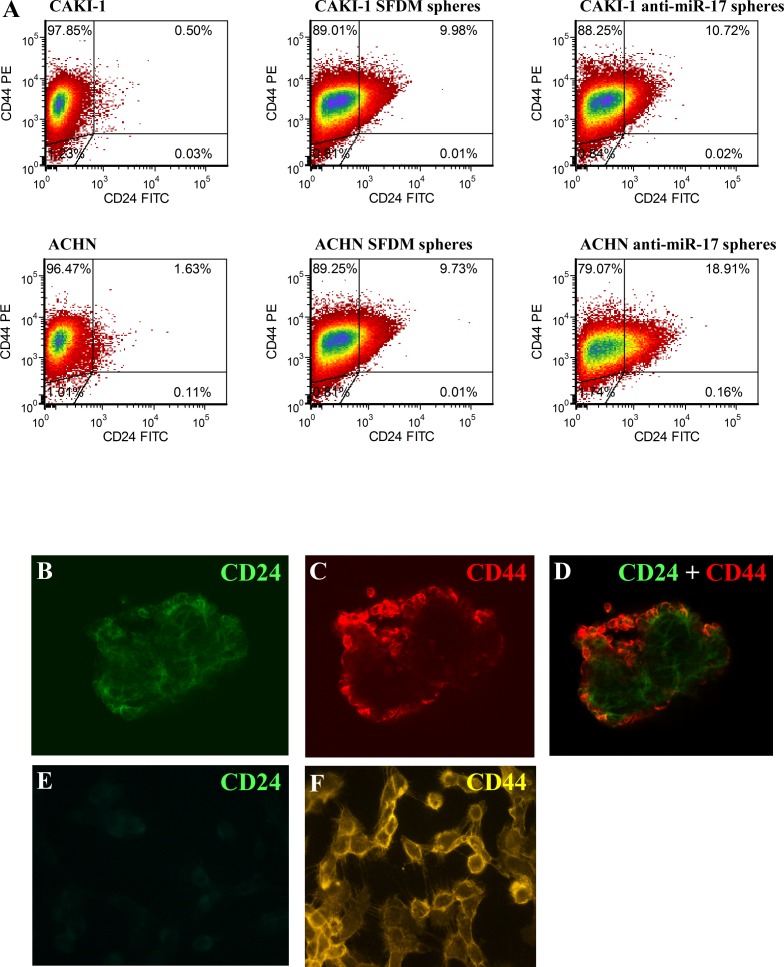
RCC cancer spheres are enriched in the CD24+ cell population (A) Parental CAKI-1 and ACHN cells and their sphere derivatives established in SFDM and or by anti-miR-17 transfection were subjected for flow cytometric analysis to detect CD24 and CD44 cell surface proteins. CD24+/CD44+ ratio was significantly higher in SFDM spheres. Spheres derived from anti-miR-17 transfection showed a further increase in the double positive cell population. Cd44 and Cd24 expression was also assessed by immunocytochemistry. ACHN spheres stained positive for CD44 and CD24 (B-D) but adherent ACHN cells appears to be CD24 negative (E-F).

To further characterize these sphere-forming cells, we examined the presence of two common cancer stem cell surface markers: CD24 and CD44 [25]. Only 0.5% of CAKI-1 parental cells stained double positive for CD44 and CD24. However, 10% of the CAKI-1 sphere cells were CD44+/CD24+. Similar results were obtained with ACHN cell, where the 1.63% of CD44+/CD24+ of the parental cells increased to 9.73% in the spheres (Figure 2A and Supplementary Table 1). We also confirmed CD24 expression in RCC spheres by confocal microscopy (Figure 2B-F). Taken together, these data support the acquisition of stem cell-like properties coupled to EMT in both ACHN and CAKI-1 spheres.

### RCC spheres exhibit increased tumorigenic ability *in vivo*

**Figure 3:**
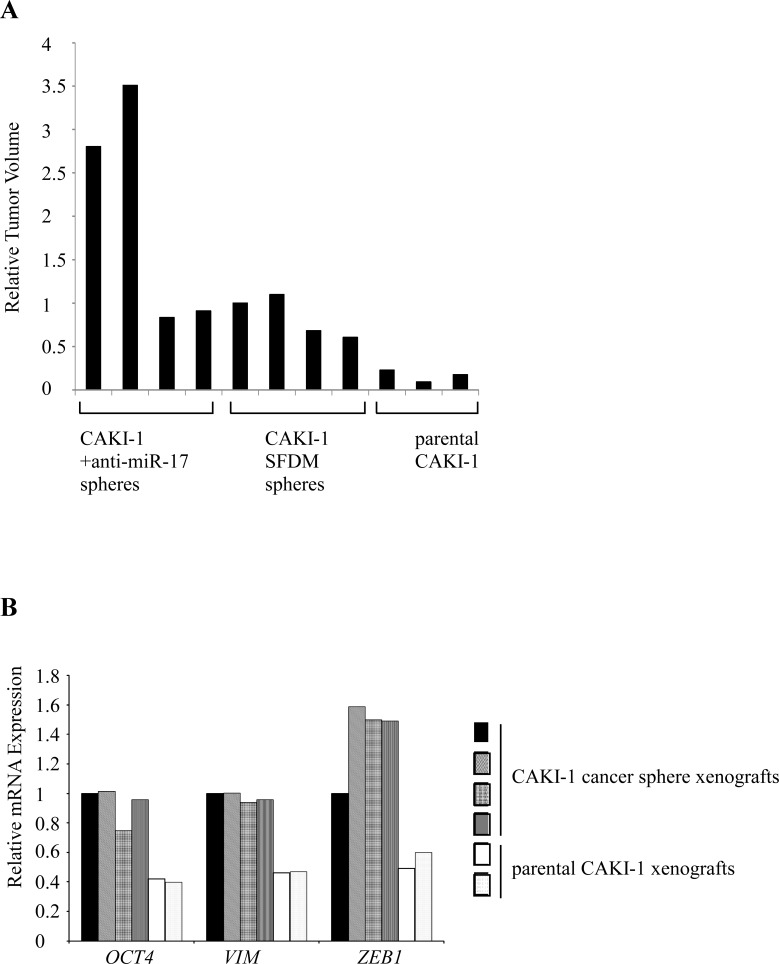
CAKI-1 cancer spheres form larger xenograft tumors with increased EMT marker expression compared to parental cells (A) CAKI-1 cancer sphere xenografts had larger volume than the parental cell-derived xenografts. The graph shows the tumor volumes compared to the CAKI-1 sphere cell xenograft. (B) RT-qPCR quantification compared *OCT4* and EMT marker expression between xenografts.

We next tested the tumorigenic potential of CAKI-1 spheres in xenograft model. 10^4^ or 10^6^ RCC sphere cells or parental cells were subcutaneously injected into NOD/SCID/γ(c)(null) (NSG) mice. Sphere-derived cells formed tumors in 4/4 mice in both dilutions. Injection of 10^4^parental CAKI-1 cells resulted in tumor formation in 3/4 mice, and all mice (4/4) developed tumors only when 10^6^ cells were injected. Tumors of the cancer sphere xenografts were significantly larger and more vascular than the parental xenografts [1037(+/−326) mm^3^] vs. [266.3(+/−78.7) mm^3^], respectively (Figure 3A and Supplementary Figure 1D). RT-qPCR analysis of the xenografts showed significant increase in both stem cell and mesenchymal markers compared to parental cells (Figure 3B).

**Figure 4:**
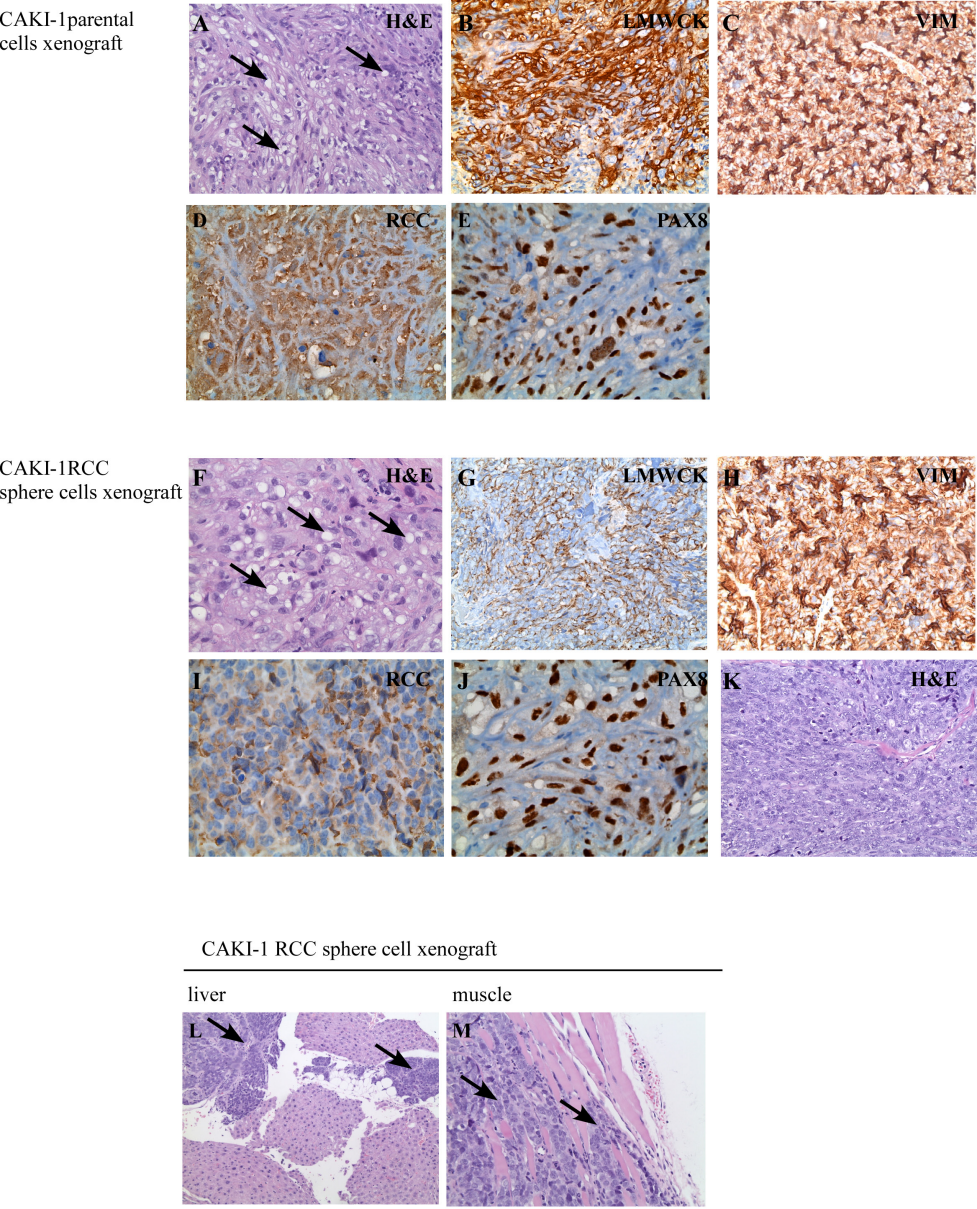
Histological features of xenograft tumors CAKI-1 parental cell xenografts developed tumors with typical RCC morphology (H & E staining) (A). Xenografts were positive for the RCC markers low molecular weight cytokeratines (LMWCK) (B), vimentin (VIM) (C), RCC (D) and PAX8 (E). CAKI-1 sphere xenografts developed tumors with typical RCC morphology (H & E staining) (F). Xenografts were positive for the RCC markers low molecular weight cytokeratines (LMWCK) (G), vimentin (VIM) (H), RCC (I) and PAX8 (J). CAKI-1 sphere xenografts also contained areas of undifferentiated, sarcomatoid RCC (H&E staining) (K). CAKI-1 sphere xenografts also developed metastatic lesions in the liver and muscle (L-M).

Hematoxylin and eosin (H&E) staining of the parental cell xenografts showed well differentiated tumors of typical “clear cell” morphology, with frequent lipid and glycogen deposition in the cytoplasm (Figure 4A). These tumors were positive for immunohistochemical markers of “renal” differentiation, including low molecular weight cytokeratin, vimentin, RCC stain, and PAX8 (Figure 4B-E). The histologic appearance of the cancer sphere xenografts recapitulated the canonical clear cell RCC morphology (Figure 4F) and stained focally positive for the same immunohistochemical markers of “renal” differentiation, as above (Figure 4G-J). However, areas with sarcomatoid (spindle cell) differentiation were also observed in the RCC sphere xenografts (Figure 4K). Sarcomatoid changes occur as a result of epithelial to mesenchymal transition, and are signs of aggressive, therapy-resistant behavior [26].

Sarcomatoid areas of cancer sphere xenografts were strongly positive for the mesenchymal marker CD44 (Supplementary Figure 2A-B), in agreement with the literature [16, 17]. Interestingly, while only moderate vascularity was observed in the differentiated RCC areas, capillaries and vascular-like spaces were prominent in the sarcomatoid part of RCC sphere xenografts. These vascular spaces were positive for the CD31 vascular endothelial marker (Supplementary Figure 2C-D). Xenografts derived from tumor spheres, but not from the parental cell line, contained laminin tracks (Supplementary Figure 2E-F), a marker for vasculogenic mimicry. The term ‘vasculogenic mimicry’ describes blood-conducting channels formed by tumor cells. RCC sphere To confirm the ability of miR-17 inhibition to induce self-renewal, we first quantified the clonogenic xenografts also formed metastatic deposits in the liver and muscle (Figure 4L-M). The sarcomatoid component stained positive for EMA and Pan-Cytokeratin, indicating its epithelial origin (Supplementary Figure 2G-H). Taken together, CAKI-1 cancer sphere cells had the potential to fully recapitulate the parental RCC tumor, to initiate mesenchymal transition leading to sarcomatoid dedifferentiation, and to induce metastatic tumors.

### miRNAs are differentially expressed in RCC cancer spheres and may contribute to their formation

**Figure 5:**
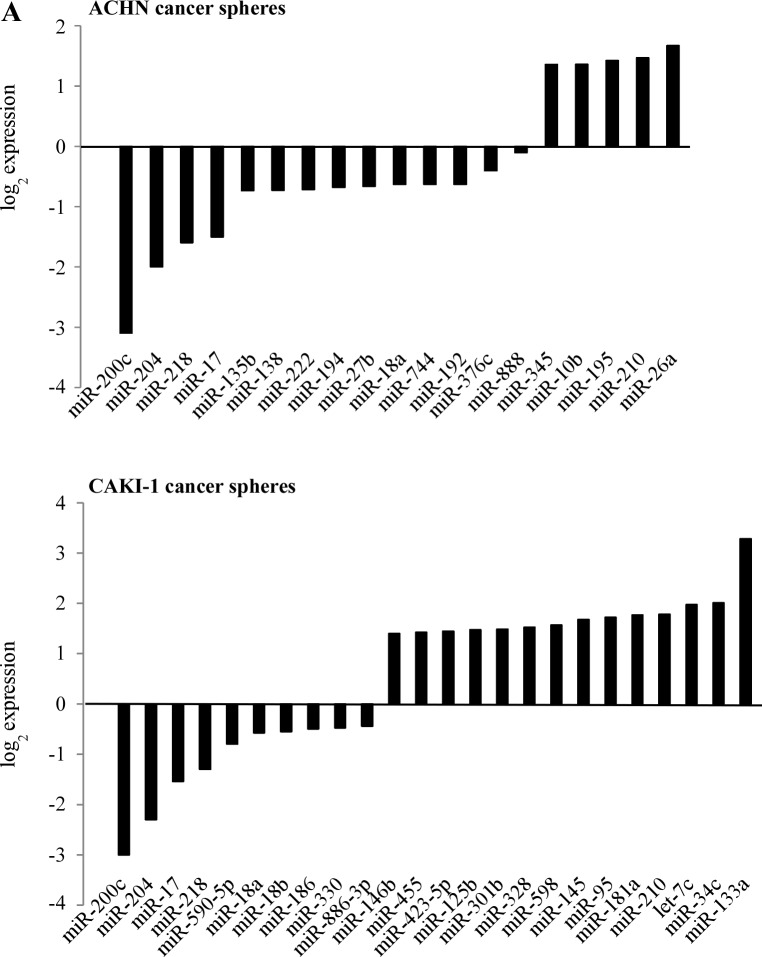

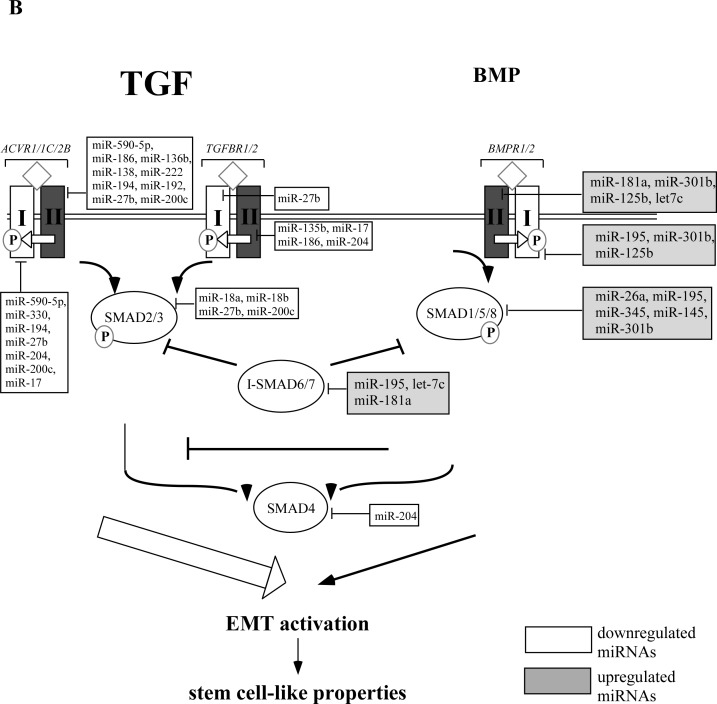
miRNAs are differentially expressed in cancer spheres and their targets are enriched in the members of the TGFβ signaling pathway (A) miRNA expression pattern of CAKI-1 and ACHN parental cells and their RCC sphere derivative was compared by RT-qPCR-based screening. (B) Targets of differentially expressed miRNAs were analyzed by miRPath and TargetScan programs. The BMPR-SMAD1/5/8-mediated branch of TGFβ pathway is targeted by several miRNAs overexpressed in RCC spheres. The TGFBR2-SMAD2/3-mediated branch of the TGFβ pathway is predicted to be targeted by several miRNAs downregulated in RCC spheres.

To understand the involvement of miRNAs in renal cancer spheres, we compared the miRNA expression pattern of the ACHN and CAKI-1 spheres with their parental cell lines. The expression of 754 human miRNAs was quantified by RT-qPCR-based screening. After eliminating miRNAs with very low expression levels (Ct value ≥ 30), 6 miRNAs showed significantly altered expression in both CAKI-1 and ACHN-derived spheres: miR-17, miR-200c, miR-204, miR-218, miR-210 and miR-18a (Figure 5A and Supplementary Figure 3). Combinatorial effect of differentially expressed miRNAs was deduced in silico by grouping their predicted targets into signaling pathways. Downregulated miRNAs were predicted to target genes that are involved in the pathogenesis of different cancers. The EMT activator ‘TGFβ signaling pathway’ and ‘WNT signaling pathway’ were also among the most significant categories (Supplementary Table 2). These pathways are documented to be dysregulated in cancer stem cells and tumor initiating cells (27). In silico analysis found that the up-regulated miRNAs are more likely to target the BMP-mediated branch of the TGFβ signaling pathway, while the downregulated miRNAs are predicted to act on the TGFBR2-mediated branch of this pathway (Figure 5B). An antagonistic effect of BMP-signaling on TGFBR2-signaling has been suggested [28]. Alternatively, the differentially expressed miRNAs may fine-tune TGFβ pathway activity [29].

**Figure 6:**
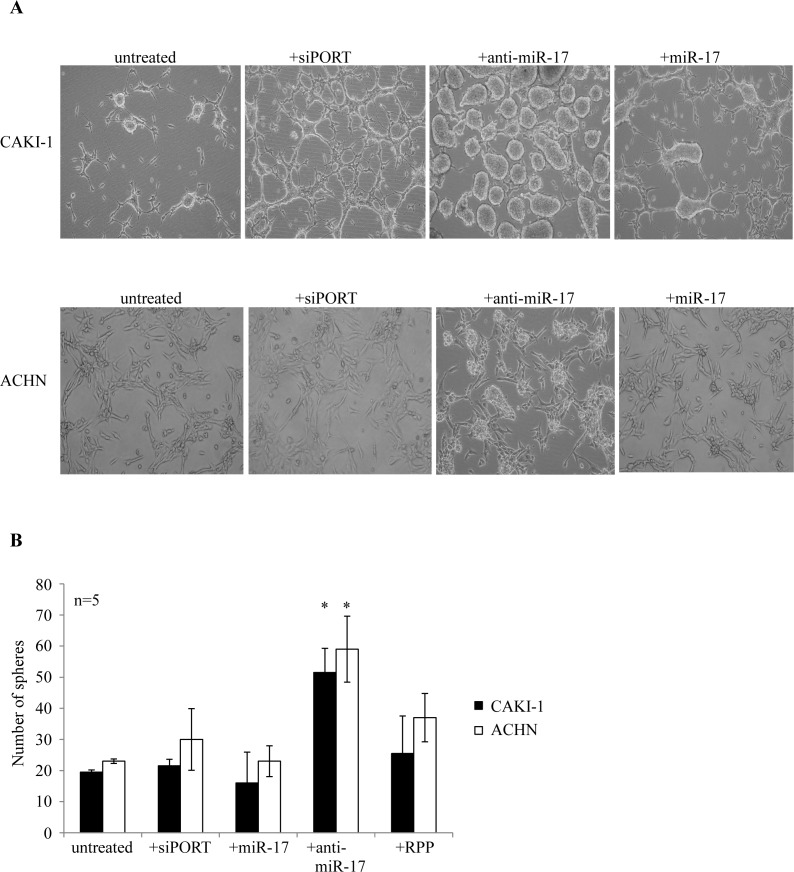
miR-17 inhibition results in formation of RCC spheres (A) CAKI-1 and ACHN cells were kept untreated in SFDM medium, were treated with the transfection agent siPORT or were transiently transfected with anti-miR-17. Sphere formation was followed for 6 days. (B) Spheres were dissociated with trypsin and secondary sphere formation capacity was assessed. RPP: random pool of miRNA precursors.

miR-17 is the best studied cancer-related miRNA [30], however, its role in cancer initiation and progression remains controversial. It is reported to regulate the TGFβ pathway [31]. miR-17 is significantly downregulated in CAKI-1 and ACHN spheres. To test a possible effect of miR-17 on RCC cancer sphere formation, we transiently transfected ccRCC cell lines with either its synthetic precursor or inhibitor. Transfection of CAKI-1 and ACHN cells with anti-miR-17 led to rapid formation of 3D spheres, which were morphologically indistinguishable from the spheres obtained in SFDM (Figure 1A). miR-17 transfection did not affect the sphere-forming efficiency. To confirm the ability of miR-17 inhibition to induce self-renewal, we first quantified the clonogenic capacity of ACHN and CAKI-1 cells upon miR-17 inhibition or treatment with transfection agent. miR-17 inhibition resulted in 2.4 fold more colonies in CAKI-1 cell line and 1.96 fold more colonies in ACHN cell line compared to the transfection agent control (Figure 6).

To have a closer insight of the molecular changes induced by miR-17 inhibition, we quantified the expression of stem cell-related transcription factors *OCT4, NANOG, KLF4* and *LIN28* in the spheres grown following miR-17 inhibition compared to parental cells. Expression levels of all stem cell markers increased significantly compared to the parental cells, similar to the spheres isolated in SFDM. Likewise, we observed elevated levels of mesenchymal markers, including *ZEB1, ZEB2*, vimentin and N-cadherin (Figure 1C-D). Next, we analyzed the expression of CD24 and CD44 cell surface proteins. Similar to the SFDM-derived spheres, we detected 21.44 and 11.6 fold increase of the double positive cell population in ACHN and CAKI-1 spheres (Figure 2).

Next we compared the in vivo tumor forming capacity of CAKI-1 spheres that resulted from anti-miR-17 and the parental cell line. Injection of 10^4^ cells induced tumor formation in 4/4 mice. The xenografts formed by the anti-miR-17 cancer spheres measured 2170 (+/− 1254) mm^3^, and were larger than the tumors formed by the SFDM-derived cancer spheres (Figure 3).

The SFDM-cancer sphere xenografts and the anti-miR-17 xenografts showed similar histology. The anti-miR-17-induced tumors were positive for RCC stain, PAX8, and focally positive for EMA, confirming their renal origin (data not shown). As is the case with the SFDM sphere-induced tumors, these tumors were positive for CD44, vimentin, laminin and diffusely positive for CD31 and CD34 staining, suggesting that the anti-miR-17 cancer sphere cells were able to recapitulate both renal and endothelial cell types of RCC.

### TGFβ pathway regulates RCC sphere formation in vitro and is a possible direct target of miR-17

**Figure 7:**
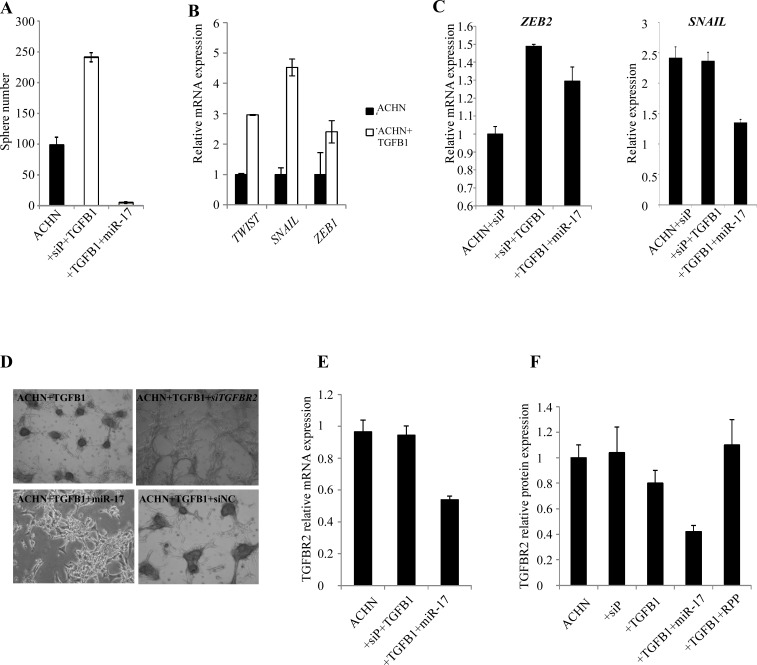

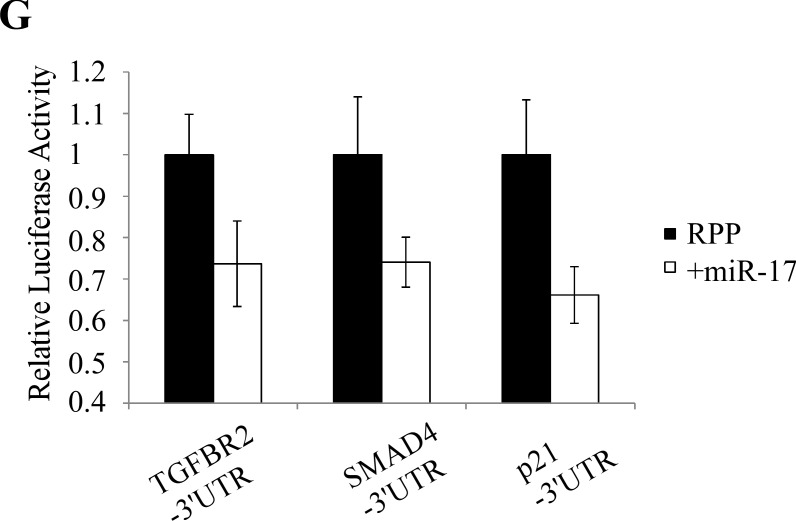

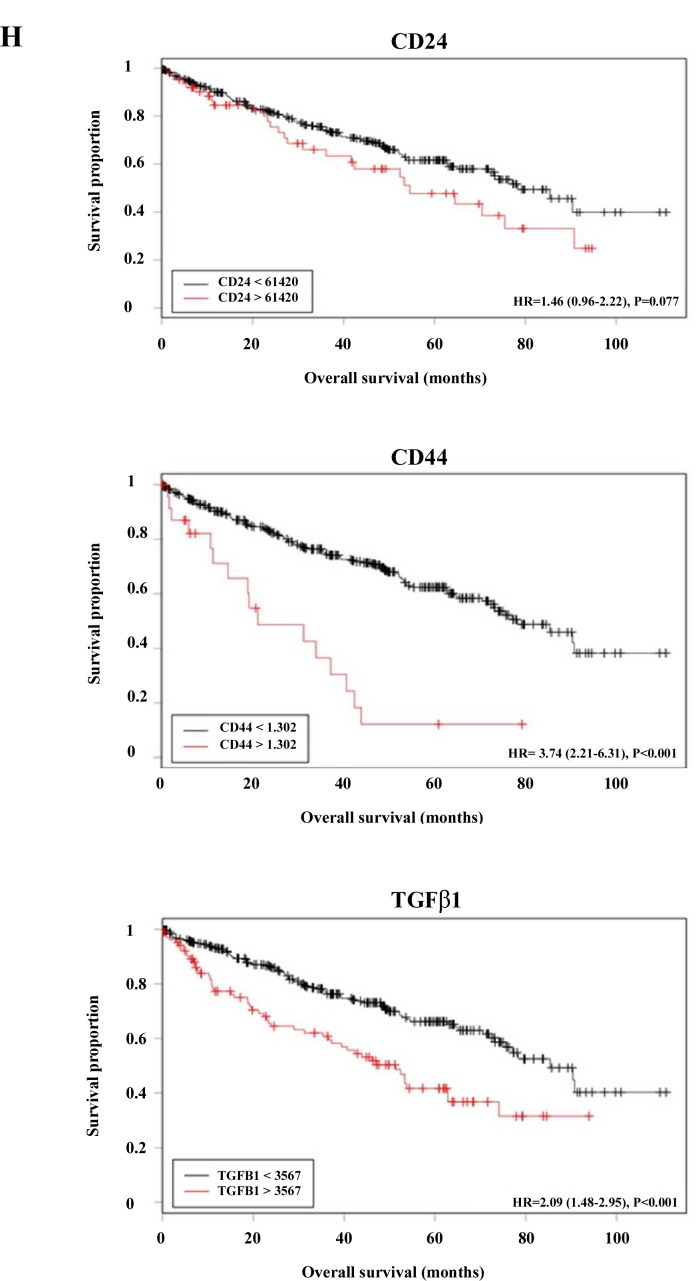
TGFβ signaling is involved in RCC formation and is regulated by miR-17 (A) TGFβ1 treatment induced sphere formation in ACHN cells. Parallel miR-17 transfection interfered with sphere formation. (B) TGFβ1 treated ACHN cells show increased expression of mesenchymal markers. (C) miR-17 transfection of TGFβ1 treated ACHN cells leads to decreased expression of *ZEB2* and *SNAIL* EMT markers. (D) Silencing of *TGFBR2* by siRNA or by miR-17 tra nsfection inhibited RCC sphere formation. miR-17 transfection leads to decreased *TGFBR2* expression at mRNA (E) and protein level (F). (F) Quantification of Western blot detecting TGFBR2 expression. (G) CAKI-1 cells were co-transfected with the luciferase constructs containing the 3′ UTRs of *TGFBR2*, *SMAD4* or *p21* and miR-17. Decreased luciferase activity indicates that *TGFBR2* is a possible direct target of miR-17. (H) Analysis of TCGA database revealed a significant increase of CD24, CD44 and TGFβ1 expression in ccRCC patients with poor survival.

To validate the effect of TGFβ-EMT axis on RCC sphere formation, ACHN cells were treated with recombinant TGFβ1. Pathway activation by TGFβ1 enhanced sphere forming capacity (Figure 7A). In parallel, we observed increased expression of the EMT markers TWIST1, SNAIL and ZEB1 (Figure 7B). To test if miR-17 interferes with the TGFβ1-induced signaling, we treated ACHN cells with TGFβ1 alone or in combination with miR-17 mimics. miR-17 mimics significantly blocked RCC sphere formation, and interfered with the expression of EMT markers (Figure 7A and 7C).

miR-17 is predicted to target *TGFBR2* at three sites, conserved across vertebrates. *TGFBR2* 3′UTR also possesses target sites for other miRNAs that were differentially expressed in RCC spheres, such as miR-204, miR-218 and the established EMT activator miR-200c. To confirm the relation between RCC sphere formation and *TGFBR2*, we activated the TGFβ pathway by TGFβ1 in ACHN cells. *TGFBR2* expression was significantly higher in cells treated with TGFβ1 (Supplementary Figure 4A). Similar increase in TGFBR2 expression was seen in the cancer spheres, compared to parental cells (Supplementary Figure 4A). We then treated cells with TGFβ1 simultaneously transfected them with a pool of four different siRNAs designed against *TGFBR2* (si*TGFBR2*), or a pool of non-targeting siRNA as control. si*TGFBR2* led to a significant reduction of TGFBR2, as expected (Supplementary Figure 4B) and interrupted RCC sphere formation (Figure 7D, Supplementary Figure 4C). These results indicate the TGFβ signaling through TGFBR2 may play an important role in the induction of self-renewing kidney spheres and that miR-17 hinders this process likely by targeting *TGFBR2*.

To assess interaction between miR-17 and *TGFBR2*, CAKI-1 cells were transfected with miR-17 mimics and (mRNA)*TGFBR2* expression was measured by RT-qPCR (Figure 7E) and Western blot analyses (Figure 7F). miR-17 transfection resulted in approximately 50% reduction in *TGFBR2* expression We further validated the miR-17- *TGFBR2* interaction using a luciferase assay. ACHN cells were co-transfected with miR-17 or a random pool of miRNA precursors (RPP) and a luciferase reporter construct containing the 3′UTR of *TGFBR2* (LS- *TGFBR2*). As a positive control, we co-transfected the cells with miR-17 and 3′UTRs of known miR-17 targets: *SMAD4* (LS-*SMAD4*) and p21 (LS-p21). Co-transfection of miR-17 and LS-*TGFBR2* or LS-*SMAD4* resulted in 26% and 24% decrease in luciferase activity, while co-transfection with LS-p21 resulted in 34% decrease (Figure 7G). These results confirm that *TGFBR2* is a direct target of miR-17 in kidney cancer.

### Increased expression of stem cell-related transcription factors is associated with worse prognosis

Using The Cancer Genome Atlas (TCGA) database, we analyzed the expression of CD24, CD44 and TGFβ1 in 511 RCC patients. Our results confirm that increased expression of these genes is associated with poor prognosis (Figure 7H). The potential prognostic utility of stem cell-related factors might be affected by the fact that CSCs represent only a small fraction of the tumor bulk.

## DISCUSSION

Current therapies aim to remove the bulk of the tumor. However, seemingly successful treatment with the approved drugs for kidney- and other cancers often end with therapeutic failure, recurrence and metastasis. The CSC model suggests that therapy resistance might be attributed to the differential drug sensitivity of the self-renewing CSC population [32], therefore a successful cancer therapy should also eradicate the stem cell population of the tumor and block the possible conversion of differentiated tumor cells to cancer stem cells. Further insight into the mechanisms by which self-renewal and differentiation of cancer stem cells is balanced will be essential for a better understanding of carcinogenesis [33].

In the current work, we isolated tumor spheres from two metastatic RCC cell lines in a medium that is commonly used to support the growth of cancer stem cells. These cancer spheres expressed significantly higher levels of stem cell-related transcription factors and mesenchymal markers and showed increased tumorigenicity in xenograft model. Though RCC spheres show several cancer stem cell characteristics, they were isolated from model ccRCC model cell lines and their composition requires more investigation. Potentially, RCC sphere cells could derive from asymmetric division of rare CSCs while the daughter cells gain proliferative ability besides keeping their stem cell characters. Alternatively, RCC spheres could derive from transient amplifying cells that accumulated mutations to allow them restoring their self-renewing ability. These cells (combining stemness with proliferating capacity) are also referred as ‘stemloids’ [34] and they represent an attractive cancer therapeutic target [35].

We provide multiple lines of evidence that the TGFβ-EMT axis is an active contributor to RCC sphere formation. First, we used miRNA expression landscape to deduce signaling pathways that operate in RCC cancer spheres. Functional clustering of the predicted targets of the differentially expressed miRNAs identified TGFβ signaling as the most promising candidate pathway. Second, RCC spheres expressed the mesenchymal marker CD44, and this is consistent with the mesenchymal cell-related surface marker expression reported in the self-renewing cell population of kidney cancer and human embryonic kidney cell line [36, 37]. Finally, TGFβ1 overexpression resulted in increased RCC sphere formation *in vitro*.

Our data suggest that the inhibition of miR-17 enhances the formation and/or stabilization of highly tumorigenic RCC spheres. miR-17 belongs to an oncogenic miRNA cluster which is essential for development and homeostasis [38]. High miR-17 expression was described in various tumor types, including osteosarcoma, colon, lung, and kidney cancers [39], suggesting an oncogenic role. However, the dual oncogenic/tumor suppressor function of miR-17 and the other miRNAs of the family have always been suspected [40]. Copy number loss of miR-17-92 cluster and the related miR-302a/b/c/d, miR-367 and miR-320 was observed in kidney cancer [41] sporadic retinoblastoma, squamous cell carcinoma of the larynx [42], breast cancer, hepatocellular carcinoma, nasopharyngeal carcinoma, liposarcoma; and was linked to lymphatic spread of breast cancer [43]. Transgenic mice overexpressing miR-17 showed overall growth retardation, smaller organs and greatly reduced hematopoietic cell lineages underlining the importance of miR-17 in the maintenance of self-renewing, multi lineage progenitor cells. Recently, miR-17-92 and the related miR-106b/miR-25 and miR-371-373 clusters were reported to control TGFβ signaling [44] through the inhibition of *SMAD*s and *TGFBR2*; while our previous results also indicate that miR-17 inhibits *SMAD4* in ccRCC cells [45].

The direct interaction between miR-17 and the TGFβ-EMT axis could explain how the inhibition of miR-17 facilitates self-renewal and the formation of cancer stem cells/tumor initiating cells. miR-17 inhibition can lead to release of its inhibitory effect on *TGFBR2* which will be then able to contribute to TGFβ pathway activation, leading to EMT and the acquisition of stem cell-like characteristics. Interestingly, elevated TGFβ activity correlated with poor disease-free survival of ccRCC [46, 47]. Oshimori et al has suggested that TGFBR signaling can antagonize BMP signaling and regulate the quiescence of hair follicle stem cells [28]. It would be interesting to fully investigate the interplay between the TGFBR and BMPR-mediated signaling and the miRNAs that showed differential expression in the RCC spheres.

Finally, we observed strong positivity for the CD31+ and CD34+ vasculo-endothelial cells in the xenografts grown from the cancer spheres, arguing that the isolated cancer spheres were indeed enriched in multipotent cancer stem cells/tumor initiating cells. Moreover, laminin positive cells indicate possible vasculogenic mimicry in the cancer sphere-derived xenografts and EMT reportedly induces their formation [47].

To conclude, we isolated RCC spheres from two kidney cancer cell lines and showed that they exhibit cancer stem cell/tumor initiating cell-like properties including the formation of self-renewing spheres in serum-free defined media, high tumorigenic capacity in xenograft model and increased expression of stem cell-specific transcription factors. We also present evidence that the activated TGFβ-EMT axis contributes to the acquisition of stem cell properties in metastatic ccRCC cell line models and this process is regulated by miRNAs. We show that the inhibition of a single miRNA, miR-17, facilitates the formation of highly tumorigenic cancer spheres in RCC cell lines and this property was sustained through several passages. Our results are in agreement with the reported role of miRNAs in stem cell maintenance and the importance of TGFβ regulated EMT in stem cell renewal.

## MATERIALS AND METHODS

### Cell line models and sphere culture conditions

ACHN and CAKI-1 RCC cell lines were purchased from ATCC (Manassas, VA). Under standard conditions, cells were grown in DMEM (glucose 4.5g/l) supplemented with L-glutamine, sodium pyruvate, 10% FBS and Penicillin/Streptomycin, in a humidified atmosphere at 37°C under 5% CO_2_. CAKI-1 cells were grown in McCoy's media, supplemented with 10% FBS, non-essential amino acids, and Penicillin/Streptomycin (Wisent Inc, St-Bruno, Canada).

Sphere culture conditions: Parental cell lines or the floating spheres obtained from transfections were plated in serum-free defined media (SFDM): low-glucose (1g/l) DMEM and supplemented with L-Glutamine, sodium pyruvate, Penicillin/Streptomycin (Wisent Inc), 20ng/ml basic FGF, 20ng/ml EGF, and B27 (Invitrogen, Grand Island, USA) using 24-well ultra-low attachment plates (Corning Inc, Tewksbury, USA). Spheres were dissociated with trypsin every 5-7 days and split to 1:3 ratio. For sphere formation assay, 500 cells/well were seeded in ultra-low attachment 96-well plate (Corning Inc) in 100 μl SFDM/well. 25μl/well SFDM was added every day for 3 weeks. Wells were photographed at 4x magnification (Nikon Eclipse TS100). Sphere number was determined by ImageJ program.

TGFβ1 treatment: ACHN cell were treated with recombinant human TGFβ1 (R&D Systems) for 3 days, final cc. 1 ng/ml.

### miRNA mimics and siRNA transfection

miRNA mimics transfections were performed in 6-well plates, using siPORT-NeoFX transfection agent (Ambion, Austin, USA) as recommended by the manufacturer. All transfections were carried out at least in three independent biological parallels, and each transfection had three technical parallels. Synthetic miR-17 precursor and inhibitor were purchased from Applied Biosystems (Foster City, USA) and were transfected at a final concentration of 30 nM. Overexpression or inhibition of miR-17 was verified by RT-qPCR quantification of its' known targets, RB1, p107 and RB2, 48 hours after transfection.

SMARTpool ON-Target *plus* siRNA or ON-Target *plus* Non-targeting Control 1 siRNA were transfected by DharmaFECT1 as directed by the manufacturer (Waltham, MA) in a final concentration of 100 nM.

### Luciferase assay

pLightSwitch 3′UTR luciferase reporter constructs for LS-TGFBR2, LS-SMAD4 and LS-p21 were purchased from SwitchGear Genomics (Menlo Park, USA). Transfections for luciferase assay were carried out in 96-well plates following the manufacturer's recommendations.

### RNA isolation and RT-qPCR analysis

Total RNA was isolated with RNeasy RNA isolation kit (Qiagen, Hilden, Germany), mature miRNAs were individually reverse-transcribed by TaqMan miRNA Reverse Transcription Kit and miRNA relative expression was quantified by specific TaqMan miRNA Assays in TaqMan Fast Universal PCR Mix. miRNA expression was normalized against RNU44 using the ΔΔCt method. mRNAs were randomly reverse-transcribed by High Capacity RNA-to-cDNA Kit and relative expression was measured by Power SYBR Green PCR Master Mix (all from Life Technologies Inc). Relative expression values were calculated using ΔΔCt method against *RPLP0* and *HPRT1* endogenous controls. All reactions were run on the Viia-7^TM^ Real-Time PCR System (Life Technologies Inc). The primer sequences used for RT-qPCR are listed in Supplementary Table 3.

### Flow cytometric analysis and immunocyto chemistry

1X 10^6^ cells were labeled with PE-conjugated anti-CD44 (Biolegend, San Diego, USA) and FITC-conjugated CD24 (BD Pharmingen, Mississauga, Canada) for 20 min, washed twice, resuspended in PBS and analyzed on a BD FACSCalibur™ platform (Beckon Dickinson, Quebec, Canada) or MACS Quant Analyzer (Miltenyi Biotec, Cologne, Germany). Data was analyzed by FCS Express (BD Bioscience) or MACS Quantify (Miltenyi Biotec) softwares using floating quadrants to enumerate negative, single-positive and double-positive populations. CD44 and CD24 double-labeling studies always included double negative and single positive staining controls for compensation. Immunocytochemistry was performed as described before. Staining was visualized by Zeiss LSM700 confocal laser scanning microscopy system or Nikon inverted microscope.

### Animals and xenograft model

The handling of mice and experimental procedures protocol were approved by the Animal Care Committee of St Michael's Hospital (Toronto, CA). 6-weeks old male NOD.Cg-*Prkdc^scid^ Il2rg^tm1Wjl^*/SzJ mice were purchased from The Jackson Laboratory (Maine, USA). Cells from ACHN, CAKI-1 parental cell lines or from the sphere derivatives were trypsinized washed twice and counted. 10^3^ or 10^6^ cells were resuspended in 100μl PBS, mixed with equal volume of Matrigel (BD Biosciences) and administered subcutaneously in the neck area. Mice were sacrificed after 6 weeks, tumors and organs (liver, lung, kidney and possible sites of invasion) were harvested and tumors were measured. Tumor volume was estimated by using the ½×(length × width)^2^ formula. Xenografts were divided into two (where possible) and were snap-frozen for RNA isolation and fixed in formalin for immunohistochemistry.

### Immunohistochemistry

Immunohistochemical analysis was performed as described before  [48]. Briefly, 4*μ*m thick sections were stained with hematoxylin and eosin, as well as CD34, CD31, laminin, Vimentin, Pax8, RCC, EMA Pan-Cytokeratine and low molecular weight cytokeratins. The primary antibodies anti-CD34, anti-CD31, anti-Pax8 and anti-Vimentin (pre-diluted; Ventana Medical Systems, Tucson, AZ, USA), anti-LMWCK (1:20; Becton Dickinson, Franklin Lakes, NJ, USA), anti-CD44 (NeoMarkers, Fremont, CA, USA) and anti-laminin (1:20; Agilent Technologies, Glostrup, Denmark) were used for staining on a Benchmark staining platform (Ventana Medical Systems), with the BMK iVIEW DAB Paraffin detection kit (Ventana Medical Systems). After drying, histologic images were obtained by ScanScope XT digital scanning system (Aperio Technologies Inc., Vista, CA) at 20x or 40x magnification. CD34 and CD31 were also used to stain mouse liver and lung and confirmed no-cross-reaction with the mouse background (data not shown).

### Western Blot Analysis

Western blot analysis was performed with anti-TGFBR2, anti-P-SMAD3 and anti-GAPDH primary antibodies (Cell Signaling), using anti-rabbit IgG HRP secondary antibody (Promega). Images were acquired and quantified on VersadocTM Imager (BioRad).

### miRNA expression screening by TLDA cards and RT-qPCR analysis

For the screening study 500ng total RNA from each specimen was reverse transcribed by Megaplex Primer Pool Human Set v2.0 A+ B (Life Technologies), using TaqMan® miRNA Reverse Transcription kit as suggested by the manufacturer. cDNA samples of individual patients were analyzed by TaqMan® Array Human MicroRNA Card Set v2.0 A+B. For validation of differential expression, the relative expression of mature miRNAs was quantified by miRNA-specific TaqMan miRNA Assays (Life Technologies). miRNA-specific reverse transcription was carried out using TaqMan miRNA Reverse Transcription Kit (Life Technologies) using 500 ng template total RNA. RT-qPCR reactions were performed with TaqMan Fast Universal PCR Mix (Life Technologies), as recommended by the manufacturer. Ectopic and endogenous miRNA levels were normalized against RNU48, and relative expression was calculated by the ΔΔCt method.

### Target Prediction and Statistical analysis

TargetScan 5.1, PicTar and TargetCombo (Union query) were used for target prediction in silico. miRPath was used for the functional clustering of miRNA targets.

Expression of CD24, CD44 and TGFβ1 was verified at mRNA level in ccRCC using the publically available ‘Level 3′ ccRCC dataset of The Cancer Genome Atlas, made available through cBio Genomics Portal. Matched gene expression and survival data for 511 ccRCC patients was available. Cut off levels were determined using Cutoff Finder software.

## SUPPLEMENTARY MATERIAL, FIGURES, TABLES



## Non-standard abbreviations

RCC: renal cell carcinoma
EMT: epithelialmesenchymal transition
OCT4: octamer-binding transcription factor
RPP; SFDM: serum-free defined media
siPORT: transfection agent, random pool of miRNA precursors
VIM: vimentin, H&E, hematoxylin-eosin staining
HPRT1: hypoxanthine phosphoribosyltransferase 1
RPLP0: ribosomal protein large, P0
TGFBR2: transforming growth factor β receptor 2
BMPR: bone morphogenic protein receptor
ACVR: activin receptor
SMAD4: mothers against decapentaplegic homolog 4
I-SMAD: inhibitor of SMADs
ZEB: zinc finger E-box binding homeobox
CML: chronic myeloid leukemia
ccRCC: clear cell renal cell carcinoma
NSCL: non-small cell lung carcinoma
