# Supplementary material for: miR-17 inhibition enhances the formation of kidney cancer spheres with stem cell/tumor initiating cell properties
Source: Oncotarget. 2014 Apr 16;6(8):5567–81. doi: 10.18632/oncotarget.1901 (PMC4467387; doi:10.18632/oncotarget.1901)
Supplement: Supplementary file 1 [file oncotarget-06-5567-s001.pdf]

miR-17 inhibition enhances the formation of kidney cancer spheres with stem cell/ tumor initiating cell properties

Supplementary Material

Supplementary Table 1: RCC spheres express CD44 and CD24. Flow cytometry analysis indicates an accumulation of CD44/CD24 double positive cell population in RCC spheres compared to their parental cell lines.

	CD24+/ CD44-	CD24-/ CD44+	CD24+/ CD44+	CD24-/ CD44-
CAKI-1	0.02 (0.01)	96.2 (2.5)	0.55 (0.06)	1.5 (0.6)
CAKI-1 SFDM spheres	0.02 (0.012)	88.0 (2.7)	**9.83 (0.1)**	0.8 (0.2)
CAKI-1 anti-miR-17 spheres	0.015 (0.01)	86.7 (3.2)	**10.5 (1.2)**	0.78 (0.4)

	CD24+/ CD44-	CD24-/ CD44+	CD24+/ CD44+	CD24-/ CD44-
ACHN	0.15	96.8 (2.3)	1.7 (0.6)	1.2 (1.1)
ACHN SFDM spheres	0.1 (0.1)	88.5 (2.1)	**9.98 (0.8)**	1.4 (0.8)
ACHN anti-miR-17 spheres	0.1 (0.15)	81.2 (2.7)	**15.2 (3.6)**	1.5 (1.2)

Supplementary Table 2: Signaling pathways predicted to be regulated by differentially expressed miRNAs in RCC spheres.

Signaling pathways regulated by all miRNAs down-regulated in RCC spheres. Bold letters indicate the pathways that also regulate epithelial-to-mesenchymal transition.

KEGG pathway	p-value
**Regulation of actin cytoskeleton**	1.16E-05
Transcriptional misregulation in cancer	1.16E-05
Neurotrophin signaling pathway	3.34E-05
**Jak-STAT signaling pathway**	0.000205
MAPK signaling pathway	0.000344
**PI3K-Akt signaling pathway**	0.000344
**Wnt signaling pathway**	0.000452
Insulin signaling pathway	0.000555
Endocytosis	0.000697
GnRH signaling pathway	0.000802

**Focal adhesion**	0.000802
Adherens junction	0.000858
Vasopressin-regulated water reabsorption	0.001345
Pathways in cancer	0.002096
Amphetamine addiction	0.003113
**Chemokine signaling pathway**	0.003268
Shigellosis	0.003484
Ubiquitin mediated proteolysis	0.00389
Dorso-ventral axis formation	0.007361
Prostate cancer	0.009496

Signaling pathways predicted to be regulated by individual miRNAs that were down-regulated in RCC spheres.

<u>Hsa-miR-200c-3p</u>

KEGG pathway	p-value
**ECM-receptor interaction**	2.26E-05
**Notch signaling pathway**	0.00169
Viral carcinogenesis	0.008868
Bacterial invasion of epithelial cells	0.008986
Prostate cancer	0.008986
Adherens junction	0.014605
Melanogenesis	0.014605
Pathways in cancer	0.020888
RNA transport	0.039975
**Wnt signaling pathway**	0.044952
**Renal cell carcinoma**	0.044952

<u>Hsa-miR-204-5p</u>

KEGG pathway	p-value
Adherens junction	1.44E-07
Colorectal cancer	1.44E-07
Endocytosis	0.001279
Pathways in cancer	0.001279
Chronic myeloid leukemia	0.001279
Pancreatic cancer	0.001402
**TGF-beta signaling pathway**	0.002071
Hepatitis B	0.002071

Apoptosis	0.002198
Chagas disease (American trypanosomiasis)	0.002497
Osteoclast differentiation	0.004201
Transcriptional misregulation in cancer	0.024475
Toxoplasmosis	0.028874
MAPK signaling pathway	0.035231
Amyotrophic lateral sclerosis (ALS)	0.035231
HTLV-I infection	0.035231
Cytokine-cytokine receptor interaction	0.047374

\

Hsa-miR-218-5p

KEGG pathway	p-value
ECM-receptor interaction	5.23E-09
Small cell lung cancer	3.74E-05
Epithelial cell signaling in Helicobacter pylori infection	0.000846
Chronic myeloid leukemia	0.00157
Amoebiasis	0.002457
Acute myeloid leukemia	0.002971
PI3K-Akt signaling pathway	0.003304
Pathways in cancer	0.003304
Transcriptional misregulation in cancer	0.00921
Prostate cancer	0.010115
mTOR signaling pathway	0.018688
Adipocytokine signaling pathway	0.028456

Hsa-miR-17-5p

KEGG pathway	p-value
Pathways in cancer	1.26E-17
Hepatitis B	5.00E-12
Colorectal cancer	3.92E-11
Bladder cancer	1.38E-10
Chronic myeloid leukemia	1.54E-09
Pancreatic cancer	3.47E-09
**TGF-beta signaling pathway**	2.72E-07
Small cell lung cancer	2.47E-06
**PI3K-Akt signaling pathway**	7.58E-06
Prostate cancer	3.14E-05
Melanoma	4.75E-05

HTLV-I infection	5.34E-05
Glioma	7.94E-05
p53 signaling pathway	0.000216
Acute myeloid leukemia	0.000514
**Focal adhesion**	0.001084
Endometrial cancer	0.001743
Cell cycle	0.002619
Transcriptional misregulation in cancer	0.002791
Non-small cell lung cancer	0.004204
Toxoplasmosis	0.008161
Thyroid cancer	0.015218
Epstein-Barr virus infection	0.020853
ErbB signaling pathway	0.025888
**Cytokine-cytokine receptor interaction**	0.025888
**Wnt signaling pathway**	0.037651
**HIF-1 signaling pathway**	0.039581
Adherens junction	0.039581

Hsa-miR-18a-5p

KEGG pathway	p-value
**TGF-beta signaling pathway**	0.001376
Endocytosis	0.004031
Colorectal cancer	0.007088
Synthesis and degradation of ketone bodies	0.029888
p53 signaling pathway	0.029888
mTOR signaling pathway	0.029888
Transcriptional misregulation in cancer	0.029888
Chronic myeloid leukemia	0.036218
Pancreatic cancer	0.041512
Pathways in cancer	0.043927
Rheumatoid arthritis	0.047965

Hsa-miR-590-5p

KEGG pathway	p-value

Colorectal cancer	0.0007
Endocytosis	0.010513
Pancreatic cancer	0.011203
Chronic myeloid leukemia	0.011203
**TGF-beta signaling pathway**	0.01121
Adherens junction	0.01121
Chagas disease (American trypanosomiasis)	0.01121
Osteoclast differentiation	0.012021
Pathways in cancer	0.012021
Transcriptional misregulation in cancer	0.029112
MAPK signaling pathway	0.03484
HTLV-I infection	0.03484
**Cytokine-cytokine receptor interaction**	0.039368

Supplementary Table 3: Sybergreen qRT-PCR primer sequences.

Klf 5’-CTGACCAGGCACTACCGTAAA

Klf 3’- CATGTGTAAGGCGAGGTGGT

Nanog 5’- CCAACATCCTGAACCTCAGCTAC

Nanog 3’- GCCTTCTGCGTCACACCATT

Oct 4 5’- TCGAGAACCGAGTGAGAGGC

Oct 4 3’- CACACTCGGACCACATCCTTC

Lin28 5’- CAGCTTCTTCTCCGAACCAA

Lin28 3’-AGCCACCTGCAAACTGCT

Snai2 5’-GCCAAACTACAGCGAACTGG

Snai2 3’- ATCCGGAAAGAGGAGAGAGG

Twist1 5’- AGACCTAGCAGAGCGACGAG

Twist 1 3’- GCAGAGGTGTGAGGATGGTG

N-cadherin 5’- TGCCAGAGTCGTTTACAGCA

N-cadherin 3’- TGTTGTTCCGGCTAATCCTC

Zeb1 5’- CGGCGCAATAACGTTACAA

Zeb1 3’- GGCAGGTCATCCTCTGGTACA

Zeb2 3’- TTCTCATTCGGCCATTTACAG

Vimentin 5’- GACAATGCGTCTCTGGCACGTCTT

Vimentin 3’- TCCTGCAGGTTCTTGGCAGCCA

Rb1 5’- AACTCTCACCTCCCATGTTG

Rb1 3’- TGCACTCCTGTTCTGACCTC

p107 5’- TCGTTCCTTGATGGCTTGTT

p107 3’- AGCGGATCACCACCTCAATA

Rbl2 5’- CTGCCTTGAGGTCGTCACTT

Rbl2 3’- AGGCCATCTTCTGCTCTAATG

Supplementary Figures

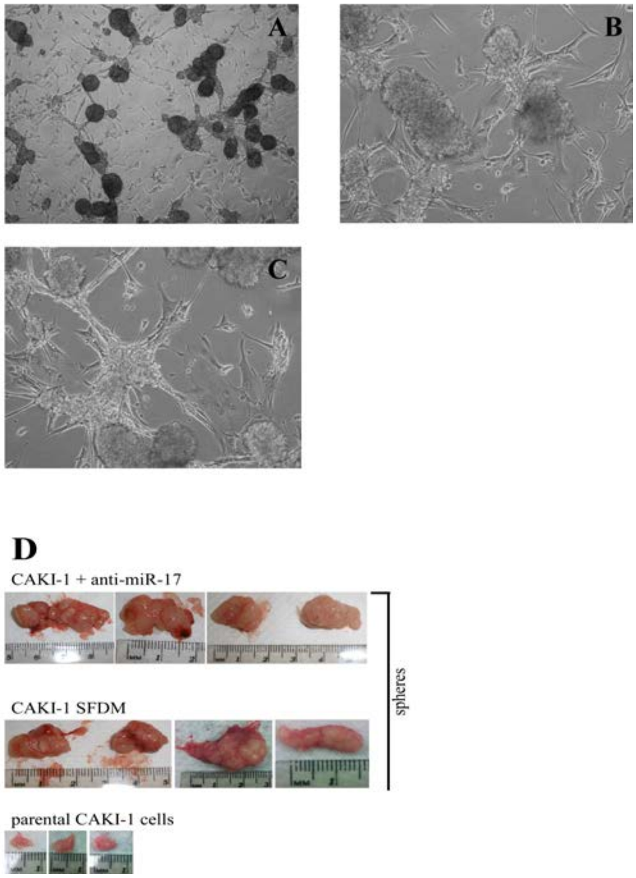

Supplementary Figure 1: (A-C) Morphological characteristics of RCC spheres. ACHN spheres were formed in SFDM and were transferred to adherent plate. Spheres quickly attached and formed subcolonies in their proximity. **(D)** Parental CAKI-1 cells or sphere cell resulting from SFDM or anti-miR-17 transfection were subcutaneously injected into NOD/SCID/γ mice and tumors were harvested after six weeks.

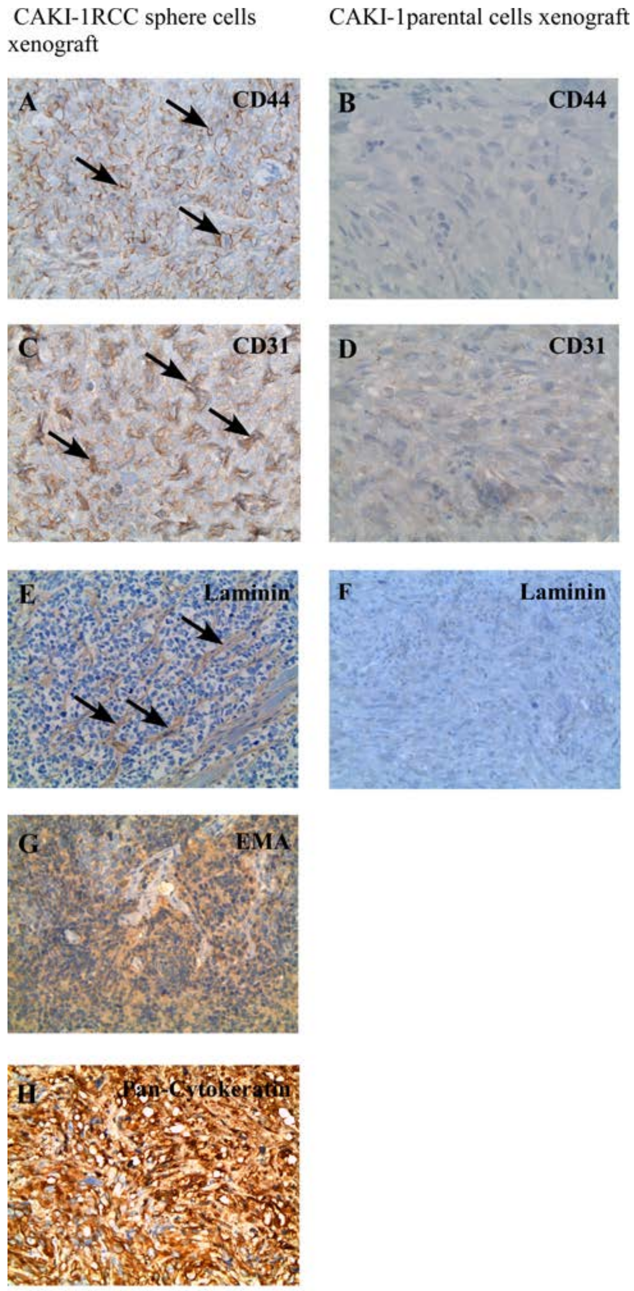

Supplementary Figure 2: Histological characteristics of the sarcomatoid areas of RCC sphere xenografts. Sarcomatoid areas of CAKI-1 sphere xenografts stain positive for CD44 **(A)**, the vascular endothelial marker CD31 **(C)**, and the vasculogenic mimicry marker laminin (**E**), compared to xenografts of the parental CAKI-1 cells **(B, D, F)** (original magnification X400). The sarcomatoid areas stained positive for EMA (**G**) and Pan-Cytokeratin **(H)**.

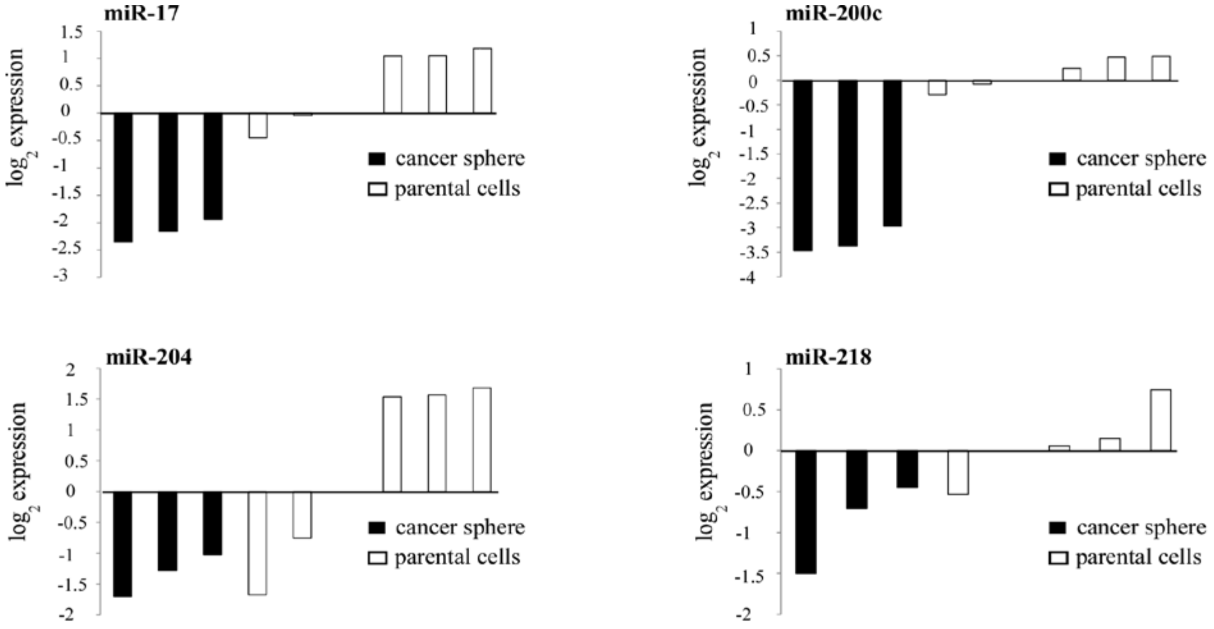

Supplementary Figure 3: miRNAs are differentially expressed in RCC spheres. Decreased expression of miR-17, miR-200c, miR-204 and miR-218 in CAKI-1 spheres was validated by comparing different sphere preparations to different stocks of CAKI-1 parental cells.

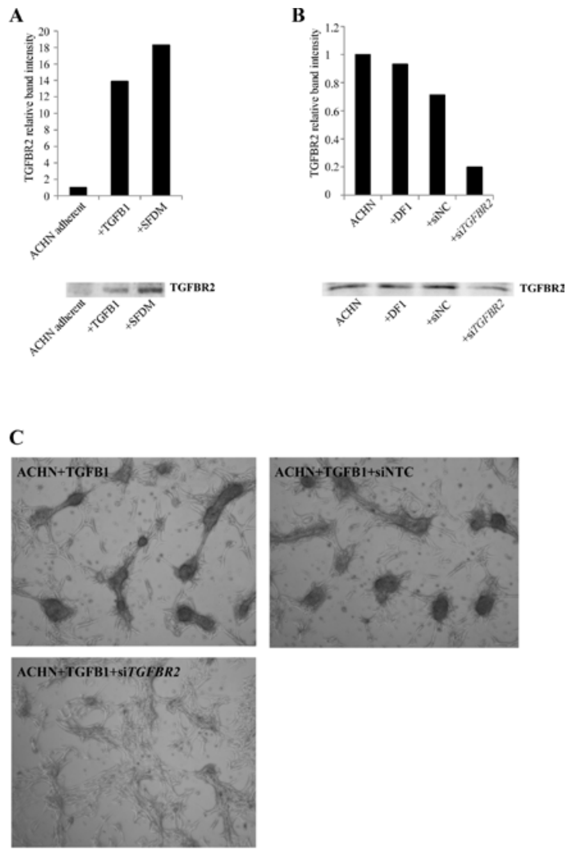

Supplementary Figure 4: *TGFBR2* expression is increased under RCC sphere supporting conditions. (A) Western blot analysis indicated increased expression of TGFBR2 upon TGFβ1 treatment or in SFDM media. **(B)** ACHN cells were transfected with a pool of four siRNAs designed against *TGFBR2*. Western blot analysis indicates reduced TGFBR level in the si*TGFBR2* transfected ACHN cells compared to cells treated with transfection agent Dharmafect (DF1) or transfected with a negative control pool of siRNAs (siNC). **(C)** TGFβ treatment of ACHN cells leads to rapid formation of colony-like structures **(left panel)**. These colonies can be propagated in 3D culture as spheres. ACHN colony formation is prevented by si*TGFBR2* transfection **(lower panel)** but not by transfection of the non-targeting pool of negative control siRNAs (siNC) **(right panel)**.
